# A novel method for diaphragm-based electrode belt position of electrical impedance tomography by ultrasound

**DOI:** 10.1186/s40560-023-00691-2

**Published:** 2023-09-25

**Authors:** Chaofu Yue, Huaiwu He, Longxiang Su, Jun Wang, Siyi Yuan, Yun Long, Zhanqi Zhao

**Affiliations:** 1 Department of Critical Care Medicine, State Key Laboratory of Complex Severe and Rare Diseases, Peking Union Medical College Hospital,Peking Union Medical College, Chinese Academy of Medical Sciences, Beijing, China; 2 Department of Intensive Care Unit, QuJing No.1 Hospital, QuJing, Yun Nan China; 3 Department of Intensive Care Unit, Shiyan People’s Hospital of Bao’an District, Shenzhen, Guangdong China; 4 Institute of Technical Medicine, Furtwangen University, VS-Schwenningen, Germany

**Keywords:** Electrical impedance tomography, Electrode belt positions, Ultrasound, Diaphragm position, Lung ventilation

## Abstract

**Background:**

This aim of study was to introduce a diaphragm-based EIT-belt placement method based on diaphragm position by ultrasound, and to evaluate the difference between diaphragm-based EIT-belt placement and conventional EIT-belt placement.

**Method:**

The diaphragm position (*L*_0_) determined by ultrasound was taken as zero reference level. The direction of headward is defined as positive, and toward feet is negative. For EIT data collection, the electrode belt was placed at 7 different levels, respectively (denoted as *L*_−2 cm_, *L*_0_, *L*_2cm_, *L*_4cm_, *L*_6cm_, *L*_8cm_, *L*_10cm_) at supine position in healthy volunteers. The diaphragm-based EIT-belt level (*L*_xcm_) was defined where highest tidal impedance variation (TV) was achieved. Subsequently, EIT measurements were conducted at diaphragm-based EIT-belt levels and traditional EIT-belt level in 50 critically ill patients under mechanical ventilation.

**Result:**

The highest TV was achieved at L_6cm_ and the smallest at *L*_−2 cm_., so the *L*_6cm_ were taken as diaphragm-based EIT-belt level by ultrasound in 8 healthy volunteers. In 23 patients, the diaphragm-based EIT-belt plane agreed with the conventional planes (4th–6th ICS), which was defined as the Agreed group. Other patients were classified to the Disagreed group (above 4th ICS). The Disagreed group has a significantly higher BMI and lower global TV at the diaphragm-based EIT-belt plane compared to the Agreed group.

**Conclusions:**

The diaphragm-based EIT-belt position by ultrasound was feasible and resulted in different belt positions compared to the conventional position in > 50% of the examined subjects, especially in patients with higher BMI. Further study is required to validate the impact on EIT images with this novel method on clinical management.

## Background

Electrical impedance tomography (EIT) is a non-invasive, radiation-free, bedside tool, which could provide information of regional lung ventilation and perfusion. EIT is commonly used in the respiratory management in critically ill patients [1–4]. EIT applies high-frequency and low-amplitude currents to the chest through 16 or 32 electrodes to obtain cross-sectional images of the lungs. The electrodes are usually embedded to a belt for clinical applications. In contrast to CT scan, EIT captures a lense-shaped volume. Studies found different plane of the EIT-belt could cause the variation of ventilation images in health volunteer and critically ill patients when the diaphragm moves into the measurement plane [5, 6]. With aim to reflect the comprehensive ventilation information, 4th–6th intercostal spaces were recommended as the standard placement of the belt [7]. However, a precise measurement plane might require diaphragm-based identification.

When EIT-belt was fixed at 4th to 6th intercostal in all conditions, the variation of diaphragm-based diaphragm position could cause improper EIT image. Kristyna et al. found the manufacturer's recommendation for electrode belt position (5th intercostal space) was unsuitable during capnoperitoneum, which caused the elevation of diaphragm [8]. Importantly, the variation of diaphragm position is quite common in critically ill patients under mechanical ventilation, such as acute respiratory distress syndrome (ARDS) with small lung volume and excessive intra-abdominal pressure, etc. A recent study indicated that the standard placement might not be ideal during titration of positive end-expiratory pressure [9]. Moreover, the diaphragm-based diaphragm position always varies in different body shape. Hence, it is relevant to build a diaphragm-based EIT-belt plane based on the diaphragm position.

Compared to the traditional EIT-belt plane of 4th–6th intercostal spaces, we speculated that the plane at the fixed distance from diaphragm position has the potential to reduce the influences caused by different diaphragm position. Moreover, EIT-plane for lung monitoring defined at the fixed distance from diaphragm position was relatively constant at the anatomical location. With the aim of reducing the impact of diaphragm-based variation of diaphragm position on EIT measurement, the present study was to introduce a diaphragm-based EIT-belt placement method based on diaphragm position by ultrasound. The difference of diaphragm-based EIT-belt placement and conventional EIT-belt placement was compared in healthy volunteers and critically ill patients under mechanical ventilation.

## Materials and methods

The prospective study was approved by the Ethics Committee of Peking Union Medical College Hospital. Written informed consent was obtained from the healthy volunteers or legally recognized family members of the patients. This study was divided into two parts.

### Part 1. Establishment of the diaphragm-based electrode belt position in healthy volunteers

**Fig.1:**
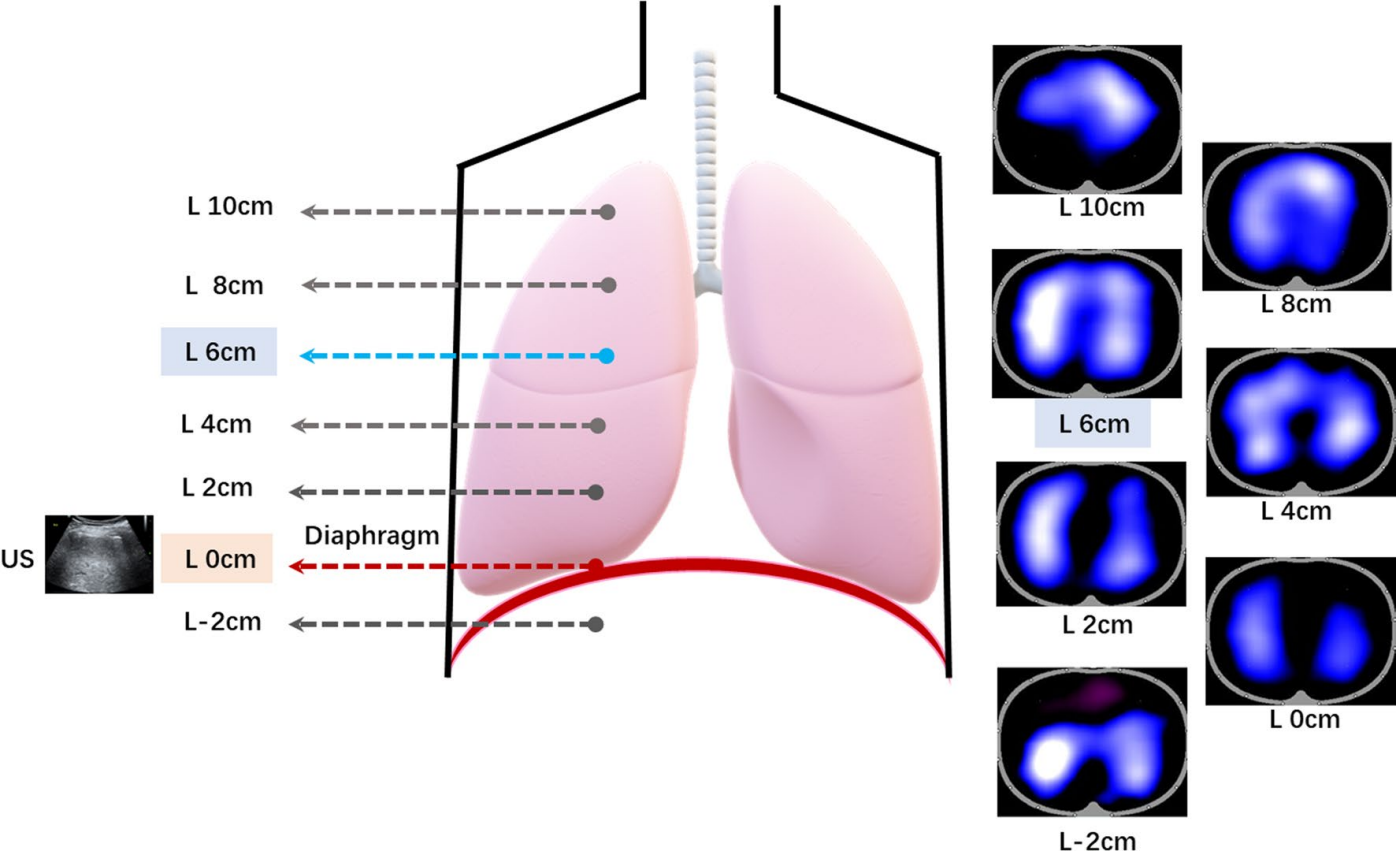
Corresponding lung EIT images of different belt positions based on the novel method in an healthy volunteer. EIT image is a 32 × 32-pixel colored matrix. Low ventilated regions are marked in dark blue and high ventilated regions in light blue to white. Purple indicates negative ΔZ

A total of 8 healthy volunteers were enrolled to determine the diaphragm-based EIT-belt placement by ultrasound. All the examinations were performed with the subjects in the supine position. The protocol was as follows:

1. Identification of diaphragm position by ultrasound With aim to identify diaphragm position, the convex abdominal probe (M9, Mindray, Shenzhen, China) was placed perpendicular to the chest wall and parallel to the ribs. The ultrasound scan started from the axillary fossa to hypochondrium at the mid-axillary line at both sides. When the superior margin of the liver was visualized at the right side, and the right diaphragm was located. The corresponding body surface position of ultrasound probe was designated as the location of the right diaphragm. When the upper margin of the spleen was displayed, and then the left diaphragm was located. The body surface position corresponding to the marker point of the ultrasound probe was designated as the position of the left diaphragm. Finally, the higher position of the left and right sides was selected as the diaphragm location (L_0_), and L_0_ was taken as zero reference level.
2. Determine diaphragm-based EIT-belt level based on the diaphragm position EIT measurements were performed with PulmoVista 500 (Dräger Medical, Lübeck, Germany). A silicone EIT-belt with 16 surface electrodes was placed around the thorax at the L_0_ defined by ultrasound. EIT measurements were performed by two investigators to ensure a rounded plane of EIT-belt. The direction of headward is defined as positive, and toward feet is negative. The electrode belt was then, respectively, placed at 7 levels (*L*_-2 cm_, *L*_0_, *L*_2cm_, *L*_4cm_, *L*_6cm_, *L*_8cm_, *L*_10cm_) with reference to *L*_0_. EIT measurements were conducted for 2 min. The height of belt was about 4 cm, and the upper edge of EIT-belt was taken as the located marker for each 2 cm movement. Moreover, the diaphragm-based EIT-belt level was identified when the highest tidal impedance variation (TV) was achieved. When the belt was moved to different levels, the re-calibration of electrical probe was performed for the measurement. Based on the novel method, corresponding lung EIT images of different belt positions was shown in Fig. 1 in an individual healthy volunteer.

### Part 2. Comparing diaphragm-based EIT-belt position and conventional EIT-belt position in critically ill patients under mechanical ventilation

ICU patients under control mechanical ventilation were screened for eligibility when the research team was available. The exclusion criteria were as follows: age < 18 years, pregnancy, body mass index over 50 kg/m^2^, ribcage malformation, and any contraindication to the use of EIT (automatic implantable cardioverter defibrillator, chest wounds limiting electrode belt placement, implantable pumps, etc.). All the examinations were performed with the subjects in the supine position.

Two-min EIT data were collected using PulmoVista500 at the 5th intercostal level (parasternal line) and the diaphragm-based EIT-belt level, respectively.

### EIT data analysis

The data were digitally filtered using a low-pass filter with a cut-off frequency of 0.67 Hz to eliminate cardiac-related impedance changes. The data were analyzed offline using customized software programmed with MATLAB R2015 (the MathWorks Inc., Natick, MA, USA). EIT images were divided into four symmetrical, non-overlapping and ventral-to-dorsal horizontal regions of interest (ROIs), ranging from the gravity-independent areas to the gravity-dependent areas, namely, the ventral (ROI1), mid-ventral (ROI2), mid-dorsal (ROI3), and dorsal (ROI4) regions. Moreover, the ventilation maps were also divided into symmetrical, non-overlapping, four cross quadrants: lower left (LL), lower right (LR), upper left (UL) and upper right (UR). TV/VT was defined as the ratio of tidal impedance variation/tidal volume.

### Statistical analysis

All data are expressed as means ± SD or medians (25th–75th percentiles) unless otherwise specified. With aim to compare difference of the two EIT-belt levels, paired data of EIT-parameters between diaphragm-based EIT-belt level and 5th ICS were compared using a paired-sample t-test or the Wilcoxon signed-rank test in critically ill patients, where applicable. With aim to clarify the potential impact factors on the difference of the two EIT-belt levels, the critically ill patients were divided into Agreed group (the diaphragm-based EIT-belt plane was in the conventional planes of 4th–6th ICS) and Disagreed group (the diaphragm-based EIT-belt plane was beyond the conventional planes of 4th–6th ICS). A Mann–Whitney test was used to compare the difference in continuous variables between disagree group and agree group in critically ill patients. With the aim to show the change trends of the EIT-related parameters among the seven EIT plane levels, the General Linear Model with Repeated Measures were performed, which is an extension of classical ANOVA that allows the handling of both fixed (different EIT-belt levels) and random (individual healthy volunteers) effects. All of the statistics were two tailed, and a *P* value < 0.05 was considered to indicate significance. The statistical analyses were performed using SPSS 24.0 (IBM, Armonk, NY, USA).

## Results

A total of eight male healthy volunteers and 50 critically ill patients with mechanical ventilation were enrolled in the present study. The demographic data are shown in Table 1.

**Table 1:** Demographic data of 8 healthy volunteer and 50 critically ill patients

Healthy volunteers (*n*)	8
Age (years)	32.25 ± 4.95
Body weight (kg)	67.25 ± 10.11
Height (cm)	169.25 ± 5.80
BMI	23.39 ± 2.57

Patients (*n*)	50
Female (*n*)	23
Age (years)	59.12 ± 14.17
Height (cm)	165.02 ± 7.09
Body weight (kg)	67.70 ± 11.92
BMI (kg/m^2^)	24.78 ± 3.40
Pts with smoking history	22
Received vasoactive drugs (*n*)	32
APACHE-II	13.42 ± 5.85
SOFA	8.3 ± 3.65
Oxygenation index	313.14 ± 125.18
PCO2 (mmHg)	39.76 ± 4.68
Respiratory rate (breaths/min)	15.94 ± 3.37
PEEP (cmH_2_O)	5.44 ± 1.07
MAP (mmHg)	92.08 ± 13.67
Major diseases	
Cardiovascular disease (*n*)	31
Digestive system (*n*)	5
Infectious diseases (*n*)	4
Urinary system diseases (*n*)	6
Gynecological diseases (*n*)	1
Pelvic tumors (*n*)	1
Respiratory diseases (*n*)	2

Height and weight were preoperative measurements upon admission. The APACHE-II score was that determined in the first 24 h after admission to the ICU. The SOFA was the highest score in the 24 h after ICU admission. The oxygenation index and PCO_2_ were collected within 2 h at the time of testing. Respiratory rate and mean arterial pressure (MAP) were recorded at the time of EIT testing

*ICU* intensive care unit, *BMI* body mass index, *APACHE-II* Acute Physiology and Chronic Health Evaluation II, *SOFA* Sequential Organ Failure Assessment, *PCO2* partial pressure of carbon dioxide, *PEEP* positive end-expiratory pressure

### Diaphragm-based EIT-belt level in the 8 healthy volunteers

The evolution of lung EIT ventilation distribution parameters during the seven levels is shown in Table 2. From *L*_−2 cm_ to *L*_+10 cm_, the TV was firstly increased and then decreased, and the highest TV was observed in *L*_6cm._ Hence, *L*_6cm_ was defined as the diaphragm-based EIT-belt plane of diaphragm position by ultrasound. From the *L*_−2 cm_ to *L*_10cm_, the ventilation distribution of ROI1% and ROI2% is gradually decreased. In contrast, the ventilation distribution of ROI3% and ROI4% is gradually increased.

**Table 2:** Variation of the EIT-related parameters at the seven different levels in healthy volunteers (*n* = 8)

Parameters	*L*_− 2 cm_	*L*_0 cm_	*L*_2 cm_	*L*_4 m_	*L*_6 cm_	*L*_8 cm_	*L*_10 cm_	*P*-value (ANOVA)
Global-TV (au)	1822 ± 970	2988 ± 733	3079 ± 863	4042 ± 1010	4957 ± 1305	2980 ± 696	1987 ± 610	< 0.0001
ROI1 Δ*Z* (au)	77 ± 104	231 ± 127	287 ± 64	435 ± 144	502 ± 104	384 ± 129	266 ± 78	< 0.0001
Percentage (%)	3.0 ± 3.0	7.5 ± 3.2	9.6 ± 1.7	11.1 ± 3.8	10.6 ± 3.0	12.9 ± 3.1	14.2 ± 4.8	< 0.0001
ROI2 Δ*Z* (au)	503 ± 307	959 ± 250	1111 ± 265	1727 ± 665	1953 ± 492	1433 ± 383	1032 ± 335	< 0.0001
Percentage (%)	28.8 ± 10.0	32.6 ± 6.1	36.6 ± 5.3	42.2 ± 8.0	40.4 ± 8	47.8 ± 4.4	52.3 ± 7.9	< 0.0001
ROI3 Δ*Z* (au)	1044 ± 540	1472 ± 394	1378 ± 481	1720 ± 790	1859 ± 603	950 ± 193	593 ± 297	< 0.0001
Percentage(%)	57.5 ± 6.9	49.3 ± 3.7	44.3 ± 4.7	41.7 ± 10.2	37.7 ± 7.1	32.2 ± 2.8	28.8 ± 9.3	< 0.0001
ROI4 Δ*Z* (au)	198 ± 131	326 ± 187	303 ± 156	372 ± 213	400 ± 262	215 ± 106	97 ± 61	0.135
Percentage(%)	10.5 ± 5.6	10.6 ± 3.9	9.4 ± 2.4	8.6 ± 3.4	7.6 ± 4.0	7.1 ± 2.6	4.7 ± 2.9	0.135
Upper right Δ*Z* (au)	264 ± 254	671 ± 236	795 ± 196	1201 ± 411	1289 ± 327	861 ± 195	615 ± 19	< 0.0001
Upper left Δ*Z* (au)	489 ± 231	311 ± 182	565 ± 169	917 ± 378	1117 ± 303	925 ± 285	660 ± 244	< 0.0001
Lower right Δ*Z* (au)	754. ± 393	1039 ± 354	948 ± 401	1189. ± 589	1221 ± 564	620 ± 187	367 ± 187	< 0.0001
Lower left Δ*Z* (au)	717 ± 243	472 ± 256	703 ± 257	877 ± 44	1002 ± 304	529 ± 133	312 ± 168	< 0.0001

### Diaphragm-based EIT-belt plane and fifth intercostal space in 50 mechanical ventilation patients

**Fig. 2:**
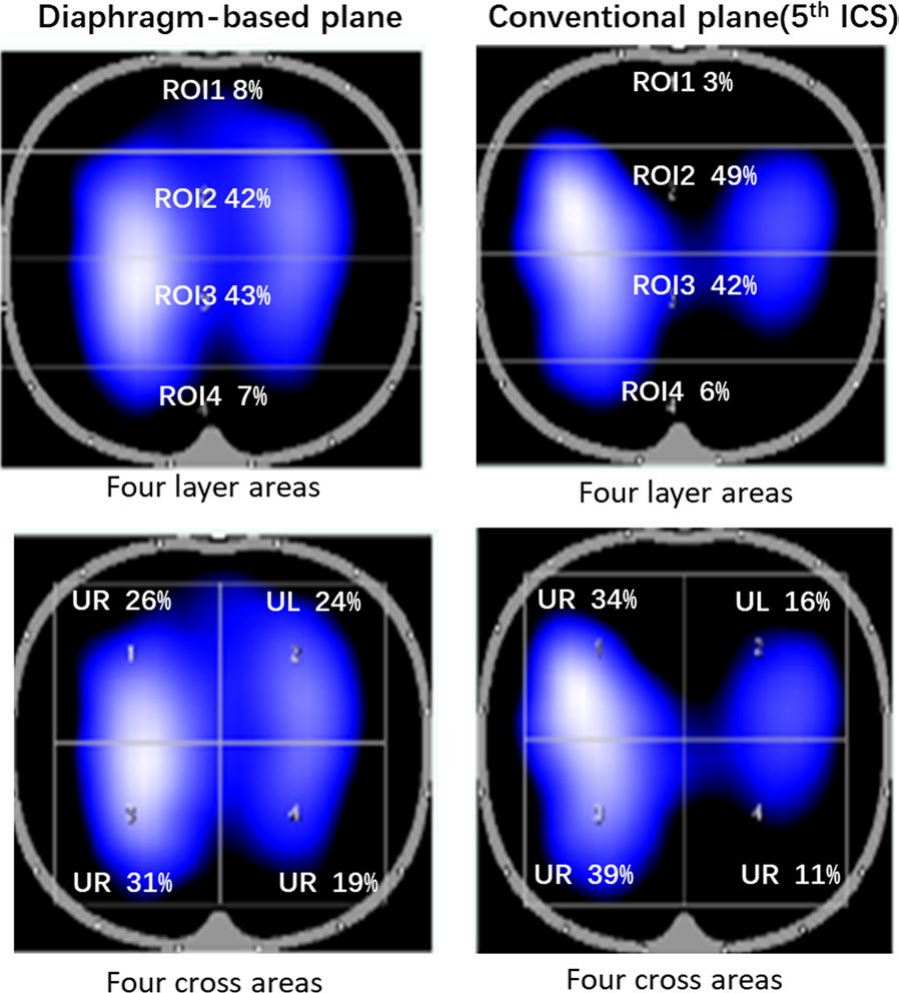
Difference of lung ventilation image in a diaphragm-based EIT-belt plane and fifth intercostal space plane. The diaphragm-based EIT-belt plane by ultrasound had a bigger area of ventilation region (eyeballing method) than 5th ICS plane

The difference of diaphragm-based EIT-belt plane and conventional EIT-belt plane in 50 patients are shown in Table 3. The diaphragm-based EIT-belt plane had a higher Glob-TV and TV/VT ratio of diaphragm-based EIT-belt plane and fifth intercostal space plane are shown in Fig. 2 in an individual patient.

**Table 3:** Difference of ventilation distribution related EIT-parameters of two levels in 50 critically ill patients

Parameters	Diaphragm-based EIT-belt level (*n* = 50)	5th ICS level (*n* = 50)	Paired differences	95%CI of the difference	*P*-value
Glob-TVΔ*Z* (au)	3082 ± 1103	2691 ± 1139	390 ± 560	231 to 549	< 0.0001
TV/VT ratio	7.12 ± 3.0	6.21 ± 3.0	1.0 ± 1.4	0.6 to 1.4	< 0.0001
ROI1 Δ*Z* (au)	365 ± 175	325 ± 171	39 ± 94	12 to 66	0.005
Percentage (%)	12 ± 5	12 ± 5	− 0.4 ± 2.7	− 1.2 to 3.6	0.291
ROI2 Δ*Z* (au)	1465 ± 541	1214 ± 500	250 ± 331	156 to 344	< 0.0001
Percentage (%)	48 ± 8	46 ± 8	1.8 ± 4.3	0.6 to 3.0	0.05
ROI3 Δ*Z* (au)	1022 ± 496	948 ± 525	74 ± 197	18 to 130	0.01
Percentage (%)	33 ± 9	34 ± 9	− 1.6 ± 4.6	− 0.3 to 0	0.018
ROI4 Δ*Z* (au)	230 ± 124	203 ± 141	27 ± 59	10 to 44	0.002
Percentage (%)	7 ± 3	7 ± 3	0.1 ± 1.5	0 to 0.1	0.363
Upper right Δ*Z* (au)	877 ± ± 383	791 ± 355	86 ± 237	19 to 154	0.013
Percentage (%)	29 ± 10	31 ± 12	− 2 to 6.5	− 4 to 0	0.027
Upper left Δ*Z* (au)	880 ± 424	692 ± 389	187 ± 242	118 to 256	< 0.0001
Percentage (%)	28 ± 9	25 ± 10	3.0 ± 6.1	1.2 to 4.7	0.01
Lower right Δ*Z* (au)	642 ± 334	589 ± 347	53 ± 201	− 4 to 110	0.70
Percentage (%)	20 ± 8	21 ± 9	− 1 ± 5.6	− 3.0 to 0.5	0.174
Lower left Δ*Z* (au)	545 ± 329	518 ± 361	27 ± 143	− 14 to 68	0.191
Percentage (%)	17 ± 7	18 ± 8	− 1.1 to 5.1	− 2.6 to 0.3	0.122

*PEEP* positive end-expiratory pressure, *TV/VT* tidal impedance variance/tidal volume, *CI* confidence interval

### Subgroup analysis of based on agreement/disagreement of conventional planes (4th–6th ICS) in 50 critically ill patients

In 23 patients, the diaphragm-based EIT-belt plane agreed with the conventional planes (4th–6th ICS), which was defined as the Agreed group. Other 27 patients were classified to the Disagreed group (above 4th ICS). There was no significant difference in the PEEP, oxygenation index, tidal volume, Glob-TV of diaphragm-based EIT-belt plane and TV/VT of diaphragm-based EIT-belt plane between agreement group and disagreement group. The disagreement group have a significantly higher BMI (26 ± 3 vs. 23 ± 2, *P* = 0.002) and lower Glob-TV of 5th ICS plane (2898 ± 1059 vs. 3214 ± 1120, *P* = 0.005) and TV/VT ratio of 5th ICS plane (5.5 ± 2.7 vs. 7.1 ± 2.9, *P* = 0.04) than the agree group (Table 4).

**Table 4:** Comparison between agreed and disagreed groups

Parameters	Agreed group (n = 23)	Disagreed group (n = 27)	P-value
Age (yrs)	55 ± 14	62 ± 14	0.085
Heart rate (bpm)	81 ± 16	89 ± 14	0.09
MAP (mmHg)	94 ± 14	90 ± 14	0.330
APACHE-II	12 ± 5	14 ± 6	0.266
BMI	23 ± 2	26 ± 3	0.002*
PEEP (cmH_2_O)	5.5 ± 1	5.4 ± 1	0.955
Oxygenation index	334 ± 135	294 ± 115	0.231
Tidal volume (ml)	467 ± 98	423 ± 73	0.092
Glob-TV_Diaphragm-based EIT-belt plane_ (AU)	3298 ± 1139	2898 ± 1059^†^	0.263
Glob-TV_5th ICS plane_ (AU)	3214 ± 1120	2246 ± 968	0.005*
TV/VT ratio _Diaphragm-based EIT-belt plane_	7.3 ± 2.9	7.1 ± 2.9^†^	0.884
TV/VT ratio _5th ICS plane_	7.1 ± 2.9	5.5 ± 2.7	0.04*

*PEEP* positive end-expiratory pressure, *TV/VT* tidal impedance variance/tidal volume, *BMI* body mass index, *AU* arbitrary unit

^*^Agreed group vs. disagreed group, *P* < 0.05

^**†**^Diaphragm-based EIT-belt plane vs. 5th ICS plane, *P* < 0.05

In the Agree group, there was no significant difference in Glob-TV, TV/VT ratio between the diaphragm-based EIT-belt plane and 5th intercostal spaces. However, the Glob-TV_Diaphragm-based EIT-belt plane_ was higher than Glob-TV_5th ICS plane_ (2898 ± 1059 vs 2247 ± 968 AU, *P* < 0.0001), and TV/VT ratio_Diaphragm-based EIT-belt plane_ also was higher than TV/VT ratio_5th ICS plane_ (7 ± 3, vs 5 ± 23, *P* < 0.001) (Table 4) in the disagree group.

## Discussion

The main finding of this study was that the diaphragm-based EIT-belt position by ultrasound was feasible. We found that tidal variation with the diaphragm-based EIT-belt position was higher than that with the conventional EIT-belt position. The optimized effect of EIT image by ultrasound is observed in the patients with higher BMI.

To objectively compare the effects of various electrode planes, we defined the “optimal” belt position was the one achieved the highest TV. Someone might argue that such criterion was arbitrary. In a previous study, the best plane of EIT-belt was defined as the highest correlation of VT and TV [6]. Since the healthy subjects in the present study were breathing spontaneously, it was not possible to track the volume changes continuously to calculate the correlation. Nevertheless, the diaphragm-based EIT-belt plane of the highest TV was a reasonable criterion: (1) the healthy subjects were asked to perform stable relaxing tidal breathing, highest TV results in highest TV/VT ratio, which implied that the EIT measurement plane covered the largest lung tissues. (2) The proposed value 6 cm over the diaphragm dome, as the diaphragm-based EIT-belt plane, within the conventional position (4th–6th ICS) in the health volunteer. (3) When the electrode belt was lower than the 6th ICS, negative TV was observed (purple area on the image). The negative TV corresponded to the opposite phase-shift movement caused from the abdominal cavity. When targeting the highest TV, the proposed method of diaphragm-based EIT-belt plane could minimize the effect of diaphragm on EIT ventilation image. Since lower lung always located at *L*-2 cm level, the ventilation was mainly distributed at dorsal region. And, upper lung was at *L*-10 cm level, the ventilation was mainly distributed at ventral region. Hence, it is easy to understand ventral distribution was increasing and dorsal distribution was decreasing when belt position was raised (Fig. 2).

Clinical studies had found the influence of various diaphragm positions on EIT lung ventilation image [5, 8]. With the aim to reduce impact of diaphragm position, the EIT-belt plane was placed at the second to fourth ICS in major upper abdominal surgery and laparoscopic gastroplasty of the obese patients [10, 11]. To resolve the impact of diaphragm-based variation of diaphragm position on EIT ventilation image, we introduced a novel method for EIT-belt location that have a fixed distance from diaphragm. Hence, the diaphragm-based EIT-belt plane by ultrasound might have a comparable tomography of the same lung than a fixed body surface position(4th–6th ICS) in the critically ill patients with various diaphragm positions. In the present study, the patients with higher BMI also had a higher diaphragm-based EIT-belt plane determined by ultrasound than the conventional plane. The obese patients always have a high location of diaphragm, so it is reasonable that a high EIT-belt location for the high BMI patients. Moreover, a higher TV and TV/VT ratio were achieved by diaphragm-based EIT-belt plane in the disagree group with higher BMI. It is supported that the novel method has potential to optimize the EIT-belt plane in the obese patients. Importantly, more than 50% patients have a different EIT-location by ultrasound than traditional EIT-belt location. In other words, traditional EIT-belt location, which might be improper, had room to improve in more than 50% patients. Since the diaphragm position is one of the main cause of inconsistent ventilation–impedance ratio, the great interest of the present study was that this innovative method might provide more consistent and precise measurements than the standard setting (4–6th ICS). These findings could be beneficial for clinical practice, which requires further investigation.

Ultrasound allows for a non-invasive, easy, accurate, reproducible, low-cost, and safe assessment of the anatomy and function of the diaphragm in mechanically ventilated patients [12, 13]. Moreover, ultrasound and EIT have much common in the respiratory failure management such as regional ventilation assessment, guide weaning of ventilator, guide PEEP titration and lung recruitment, etc. [1, 14–16]. Moreover, it is relatively easy for intensivists to learn the skill of how to find the diaphragm location. Combined ultrasound and EIT could enhance the ability for the respiratory management [17].

There are serval limitations of this study. (1) Since all healthy volunteers were male, the gender could cause the selection bias. (2) Since the lung image of diaphragm-based EIT-belt plane could be improved in the disagree group, other EIT-related parameters such as global inhomogeneity index, center of ventilation, regional ventilation delay and pendelluft parameters might be impacted [4, 18]. Further study is required to validate the impact degree of diaphragm-based EIT-belt other EIT-related parameters. (3) The best EIT-belt position varies and controversial in different aims condition. Electrode placement at 5th intercostal space might not be ideal for every diaphragm-based during EIT measurement during PEEP titration [9]. The mentioned diaphragm-based EIT-belt position by ultrasound requires validation in the titrations of PEEP and tidal volume. (4) Since EIT measurement plane is covering part of the lungs, not the entire lungs, we hypothesized that the larger the voxel EIT measurement covers, the better the electrode placement is. Nevertheless, the proposed method, defined by the highest tidal impedance variation in healthy volunteers, has inherent limitations in its reliability. Hence, the hypothesis requires further study. (5) Most of the enrolled patients was not with severe respiratory failure. Hence, this novel way of EIT electrodes placement based on diaphragm position better reflect the ventilation for in critically patients with mild respiratory failure. Interestingly, this improvement was mainly in the patients with high BMI who always had a high diaphragm position in the present study. Hence, this similar effect by novel method might be also present in patients with severe respiratory failure patients who always had a small lung and high diaphragm position. Further study is required to validate the impact of the novel method on the management of severe respiratory failure based on the lung EIT image.

## Conclusion

The diaphragm-based EIT-belt position by ultrasound was feasible and resulted in different belt positions compared to the conventional position in > 50% of the examined subjects. Further study is required to validate the impact of this novel method on clinical management.

## Data Availability

The datasets used and/or analyzed during the current study are available from the corresponding author on reasonable request.

## Abbreviations

AU: Arbitrary unit
APACHE-II: Acute Physiology and Chronic Health Evaluation II
BMI: Body mass index
EIT: Electrical impedance tomography
ICU: Intensive care unit
SOFA: Sequential Organ Failure Assessment
PCO_2_: Partial pressure of carbon dioxide
PEEP: Positive end-expiratory pressure
